# Essay *on* Gun-Shot Wounds

**Published:** 1813-12

**Authors:** M. Richerand


*For the Medical and Physical Journal.*

ESSAY *on* GUN-SHOT WOUNDS, *by* M. RICHERAND; *with additional* OBSERVATIONS *by the* TRANSLATOR.

(*Continued from p.* 383.)

DILATATION is not useful in wounds of parts not very fleshy, such as the cranium, the lower part of the leg, the foot, the wrist, and the hand. The great number of nerves and tendons in the parts last mentioned, renders every incision dangerous. We have no reason to apprehend excessive swelling in these parts, except, perhaps, in the palm of the hand, where the muscles are pretty numerous, and of some thickness. The extraction of foreign bodies is easy in the parts just mentioned. A soldier of the Parisian guard received a ball in the back of the hand; it was fixed in the space between the third and fourth metacarpal bones; being seized with a pair of dissecting forceps, it was easily extracted. The wound was not enlarged, but simply covered with a pledget of cerate; it got well without difficulty. We may therefore consider dilatation as useless in slightly fleshy parts, where the swelling is of course very circumscribed; it is dangerous, whatever may be the part wounded, when there is torpor; the solids, whose vital properties are enfeebled, would fall into a total relaxation, and gangrene would be the inevitable consequence.

Dilatation is *positively* indicated, it is indispensable only in cases where the limb has been traversed by a ball, in the most fleshy part, in a place where the muscles are covered by an aponeurosis, more or less thick. Suppose for a moment that the thigh has been pierced in its most fleshy part, without injury to the femoral artery or bone. The inflammatory swelling which must inevitably supervene, will at least double the bulk of the muscular mass, and the aponeurotic covering will resist the swelling; the pain which will result from the swelling, together with the humoral infiltration, will infallibly bring on gangrene. Dilatation is indicated to prevent a dangerous strangulation; we should enlarge the orifices of the wound, not for the purpose of changing its round form, which the old surgeons thought extremely pernicious, but to relax the aponeurosis *fascia lata*.

To perform this dilatation, we employ a probe-pointed bistory; bistory; the fore finger is to serve for its conductor. The aponeurosis itself must be slit for some inches in extent; and in order that the muscles should not form a muscular hernia through this simple longitudinal incision, we cut the fascia across, or even in various directions, if it is judged expedient.* We must unbridle the whole extent of the wound, deeply if possible, always avoiding those places where anatomy teaches us that important nerves and blood-vessels are situated. For this purpose, a bistory is to be employed with a straight and long blade, terminated by a button similar to that of Pott's probe-pointed bistory: it should be made to cut by pressing on its back with the fore finger of the left hand, which is preferable to all other directors. When the wound is so far unbridled that the muscles cannot be confined by the aponeurosis, in the swelling, which is to take place, is it necessary to pass a seton through the passage of the wound?

Many practitioners advise it, and employ it, as they say, to favor the suppuration of the wound, and the separation of the sloughs. But ought we not to consider the seton as a foreign body, whose presence increases the irritation and the

---

* M. Richerand's rule for dilating gun-shot wounds seems too limited and yet too general. It is too limited, because it confines dilatation to a limb, or to the fascia of a limb, whereas it may be proper in other parts of the body, which are suffering from inflammatory tension. It is too general, because it directs dilatation in all cases where a limb has been shot through. Mr. Hunter tells us it is impossible to state what wound ought, and what ought not to be opened;' but that we must convince ourselves of the necessity of dilatation from the circumstances of the case, and see clearly that some good is to be done by it. He prefers dilating after the first inflammation has subsided. In Mr. Chevalier's admirable treatise on gun-shot wounds, nearly the same principles are laid down. We may, perhaps, venture to state, 1st. *That any part may be dilated, which, by its inflammatory tension, produces dangerous constitutional irritation, provided that the structure of the part does not prohibit.* 2d. *That the time for dilatation is when inflammatory tension exists, and when, of course, the utility of an operation is sufficiently conspicuous.* M. Richerand deserves the reputation of restricting the dilating practice within narrow bounds, compared to what his immediate predecessors directed. M. Percy would have the wound dilated upward and downward, not only through the external parts, but the whole depth of the wound; to effect which it would sometimes be necessary, in a muscular patient, to open the limb from one end to the other. The fascia must be hacked like a saw *(dentelé)*, and if the bone is shattered, all broken pieces are to be fairly exposed, and some portion of the sound bone must also be uncovered!

 inflammatory inflammatory swelling? The seton is never changed without producing great pain, especially when a nervous cord runs near the wound. The sloughs separate when once the suppuration is well established. The seton is then dangerous in many cases, and when it does no harm, it is at least useless.

To chance, and not to his genius, did Ambrose Paré owe the useful discovery of the true method of treating gun-shot wounds. The surgeons of his day, ignorantly cruel, applied spirituous and caustic applications to these wounds. Paré, when employed in the French army at the siege of Turin, himself followed this murderous practice; but having exhausted the store of remedies commonly employed, he was compelled to use simple digestive applications, and found his patients much better under the use of them, than when treated by the old method.

After the extraction of foreign bodies, and properly unbridling the wound, gun-shot wounds require the same treatment as common contused ones: the application of pledgets of lint, covered with simple digestive ointment, together with spirituous and resolvent fomentations for the first twenty-four hours; after which, emollient cataplasms are to be applied over the lint.* As we must expect an inflammatory swelling, proportionate to the violence of the contusion, a copious bleeding is indicated, when the subject is young, vigorous, and has not experienced too violent a shock. If there should be a general stupor, or even if it be merely local, we must abstain from bleeding, and employ tonic instead of antiphlogistic remedies.

All practitioners who have written on the treatment of gun-shot wounds, concur in declaring the utility of emetics, administered on the very day of the accident, or on the day following, before the appearance of inflammatory symptoms. This practice is particularly advantageous in armies, where, from the use of bad food, and inevitable irregularities in diet, the alimentary passages are crowded with impurities. Lamartinière, in a memoir inserted among those of the Academy, has particularly insisted upon this evacuation, for preventing the traumatic fever from degenerating into one of a bilious or putrid character. This fever kindles up, the wounded part swells, suppuration takes place through the passage of the wound, detaches and sweeps away the sloughs which cover its surface; after the complete separation of

* There seems to be no satisfactory reason why poultices should not be applied in the first instance.

the slough, the wound is reduced to the condition of a common contused wound, and requires the same treatment.

We have hitherto supposed the cure not to be retarded by any particular accident: it is, nevertheless, exposed to all those which are capable of retarding the cicatrization of suppurating wounds. Sometimes also a hemorrhage takes place on the separation of the slough. The well educated surgeon will foresee this accident from the relation of the wound to the situation of important arteries; he will place an intelligent assistant near the patient, with instructions how to check the hemorrhage, till effectual aid can be given.

The wounds of fire-arms, complicated with fractures of bone, are much more dangerous than those we have hitherto spoken of. A greater or less commotion always accompanies these fractures, which are called comminutive, because the bone is broken into a number of fragments. Musquet balls less frequently produce these effects than cannon balls, pieces of bomb, and other large bodies. In sea fights, there are few slight wounds; the cannon balls dismasting the ships, the sailors are crushed by the weight of the spars; the splinters of wood, separated from the body of the vessel, are thrown with violence upon the combatants, and fracture their limbs, unless they completely tear them away. What is to be done under such circumstances? Is amputation proper in all cases of comminutive fractures with wound, and excessive contusion of the soft parts, whatever be the cause which produces them?

There was a time when a much greater number of amputations were performed in the armies of the other nations of Europe than in the French army. This practice, though dictated by an inhuman policy, was the most advantageous, according to the opinion of Bilguer, surgeon-general of the armies of the King of Prussia. According to him, amputation is rarely indicated, and we ought almost never to have recourse to it. The dissertation in which he unfolds these principles, was the subject of such scandal in France, that Lamartinière, then at the head of French surgery, thought it his duty to refute it in a memoir, which is inserted among those of the Academy of Surgery. It was suspected that Bilguer had accommodated his doctrine to the views of the great Frederic, who, being the king of a poor country, was not fond of multiplying invalids at the expense of the state.

It might seem that in cases where a limb is entirely carried away by a ball, amputation would be unnecessary; but this is one of the cases in which the necessity for performing the operation is best established. How could a wound get well in which the flesh is torn to shreds, the bone splintered, and the whole limb disorganised? How long should we have to wait the separation of the sloughs? What an enormous suppuration would take place in the midst of so much disorder? The fractured bones have received so violent a shock, as perhaps to affect the articulation; the splintered part may extend into it. If the patient escapes the accidents which first occur, will the cicatrization of such an uneven surface be possible, and what firmness would the cicatrix have after it formed? All these considerations must decide us to practise amputation upon the spot, on limbs carried away by a cannon-ball, or by any other body thrown with great violence. The operation is to be performed at some fingers' breadth above the wound, unless we have reason to suspect that the fracture extends to the joint above. In case a ball had carried away the foot at two inches above the ankle, perhaps it might be better to amputate the thigh, than to cut off the leg below the knee. The same would be proper in the arm. If the arm should be carried off near its upper part, the shoulder joint must be amputated. Instead of amputating the hip joint, the os femoris may be sawed off near the joint. The end proposed in all these operations is to substitute, for a torn and horribly contused limb, a simple wound, whose even surface is capable of a quick and regular union.

A second case requiring amputation, is when the limb is so much injured, as to be threatened with inevitable gangrene. If the bone is splintered into a great number of pieces, the flesh excessively bruised and reduced by contusion almost to a jelly, and the solids confounded with the extravasated fluids, the mortification of the limb is then certain, and amputation must be practised on the spot, before the storm of inflammatory accidents commences, and a burning fever is kindled.

If the favorable moment has been lost, or if, having wrongly judged it possible to save the limb, the wounded parts sphacelate, yet the patient resists the ravages of disease, we must then amputate at the boundary line between the living and dead parts, waiting, however, till that line be decidedly marked.

A fourth case for amputation is, when the inflammatory swelling, having been happily combated by bleeding, and an antiphlogistic regimen, terminates in snch a copious and continued suppuration, that the life of the patient is threatened by a hectic.

Surgeons have been divided as to the propriety of amputating on the field of battle. It seems to be proper when the wounded man is to be immediately transported to a distant hospital. The difficulty of transportation; the inconvenience of the waggons, in which the patients, lying in heaps, are exposed to the most painful jars, the splinters of bone continually penetrating deeper from the motion of the carriage, thus carrying the laceration and contusion, already great, to the utmost extreme, while the patient is ill protected against the external air and storms, all impress on us the necessity of immediate amputation, to save the patient from cruel tortures, and an agonizing death. It is true that amputation at the moment the system is suffering the general commotion, occasioned by the shock, does not succeed so often, as in cases where the necessity is brought on by consequent accidents. But although this be admitted, it is not probable that so large a proportion would be saved by deferring the operation, because they would fall sacrifices to fever, inflammation, gangrene, &c.*

What method of treatment is to be pursued in cases of gun-shot wounds, where the injury is not great enough to render amputation necessary, and in which, however, the derangement is considerable? The incisions proper for unbridling, for discharging extravasated fluids, as well as for facilitating the search and extraction of foreign bodies, having been practised in the manner pointed out above, we must place the wounded limb on a bolster, made of the husks of oats; a splint-cloth is to cover the bolster, and upon this splint-cloth we are to place the separate strips of the bandage of Scultetus, or many tailed bandage; then a certain number of long compresses. All this apparatus is to be wet with camphorated spirit, or some other discutient. The wound is to be dressed with lint, the pledgets of which are covered with some relaxing substance, such as common cerate. Over these pledgets we apply the compresses, then the strips of bandage, as in any other compound fracture. Three bolsters of the husks of oats, and upon these bolsters three splints, are to be applied, one before and the two others laterally, the two last having been first rolled in the splint cloth, so that there should remain only room sufficient to place the bolster. All these are to be tied moderately tight by a sufficient number of bands placed over the splints. The wounded limb is then to be laid on a pillow so fixed as to form an inclined plane toward the body. In this way we favor the return of the fluids, which is often difficult, on

* Mr. Hunter states it as the result of experience, that few have done well who had their limbs cut off on the field of battle; while a much greater proportion have done well, in similar cases, who were allowed to go on till the first inflammation was over, and underwent amputation afterwards.

account account of the shock produced by the commotion, and we have less to fear from gangrene, by the stagnation of the fluids.

One or two bleedings are to be performed immediately, provided the subject is young, vigorous, and has not lost much blood, which is commonly the case, for the surface of the wound, reduced to a slough, is dry, unless a large vessel has been wounded. In case of commotion and stupor, we must abstain from bleeding, and prescribe a generous cordial by spoonfuls, together with wine and other tonics.* Next we are, as before stated, to give an emetic, and keep the bowels open by gentle purgatives. This practice is good, not only in gun-shot wounds, but in all cases of compound fractures among the lower class of people, who are generally loaded with indigested food.

In the first twenty-four hours after the injury, the swelling and inflammatory fever commences. We must then apply poultices to the limb, and substitute a fomentation of marsh-mallows, or other relaxing herb, for the resolvents first applied.† The patient must be dieted; he must have acidulous, cooling, and diluting drinks, according to his taste and the season of the year.

If the inflammation terminates in gangrene, we must amputate, with the precautions already mentioned. If in suppuration, the quantity of pus will be proportioned to the extent and importance of the injury. The sloughs separate, the pus removes them, the wound cleanses, and the splinters re-unite to the bone, when they have been partially separated.

The disease now makes rapid progress towards the cure; but, in the greater number of cases, the termination is not so favorable. The quantity of pus, instead of diminishing, increases; and instead of being white and inodorous, as at first, it becomes sanious, fetid, and green-colored. Its abundance is such as that in spite of the closest dressing, and the most

---

* Local bleeding by leeches is recommended by the best authors, when the inflammatory swelling is great.

† Mr. Chevalier points out another case in which it is important to avoid bleeding. After the occurrence of profuse hemorrhage from gun-shot wounds, or a too free use of the lancet, the pulse becomes full apparently, and appears to demand a repetition of bleeding. But the apparent fulness of the stroke arises only from diminished tone and resistance in the coats of the artery, in consequence of weakness. They are dilated by a slighter impulse of the heart than usual. If the surgeon, misled by this pulse, takes away more blood, the patient will certainly die in a few hours.

methodical methodical compression, it is absorbed and carried into the mass of the blood, where its presence excites the purulent hectic fever. The fragments of bone, being bathed in pus, do not consolidate; local sweats and colliquative diarrhœa bring on a marasmus, which carries off the patient in a few weeks. When the first symptoms of hectic appear, we must combat them by the internal use of tonics, as has been mentioned in speaking of suppuration. But when in spite of these the colliquative diarrhœa occurs, we must haste to save the patient by the amputation of the limb. The state of weakness is, as Bell remarks, favorable to amputation; but we are not to wait, as this author recommends, till the strength of the patient is exhausted.

When particular organs are wounded, the treatment will be modified, according to the nature of those organs and the extent of the wound. This will easily be done by a surgeon who has correct ideas of anatomy and pathology.*

* New England Medical Journal, October 1812.

